# Implementation of the Ogata flow cytometric scoring system in routine diagnostics of myelodysplastic syndrome

**DOI:** 10.1002/hsr2.90

**Published:** 2018-09-26

**Authors:** Sara Maj Hyldig Matzen, Klas Kræsten Raaschou‐Jensen, Klaus Kallenbach

**Affiliations:** ^1^ Department of Clinical Biochemistry Zealand University Hospital Roskilde Denmark; ^2^ Department of Haematology Zealand University Hospital Roskilde Denmark; ^3^ Department of Haematology Odense University Hospital Odense Denmark; ^4^ Department of Clinical Pathology Zealand University Hospital Roskilde Denmark; ^5^ Department of Pathology, Rigshospitalet Copenhagen University Hospital Copenhagen Denmark

**Keywords:** CD34, FCSS, flow cytometry, MDS, pitfalls

## Abstract

**Background and Aims:**

Compiling evidence has emerged for the relevance of flow cytometric assessment as a valuable part of the diagnostic work‐up of myelodysplastic syndrome (MDS). This study aimed at evaluating the implementation of a simple flow cytometric scoring system (FCSS), the Ogata score, in a routine diagnostic laboratory.

**Methods:**

A total of 35 patient samples with a clinical suspicion of MDS were retrospectively assessed using the FCSS. The accuracy of the FCSS was evaluated on the basis of the final diagnoses of the patients.

**Results:**

The final diagnoses included 17 MDS, 4 other myeloid cancers, and 14 reactive changes. Thirty‐two of 35 (91%) were correctly scored by the FCSS. All 3 incorrect scores were from samples classified as “other myeloid cancers.” Of the initial pathological evaluation of the bone marrows, 20% were inconclusive or incorrect. All inconclusive samples were correctly scored using the FCSS.

**Conclusion:**

The FCSS evaluated here has high accuracy and low complexity. Cases with inconclusive pathological evaluation will especially potentially benefit from adding the Ogata score to the diagnostic work‐up. The system will be feasible to implement in most flow cytometry laboratories without the need for supplemental antibody panels. It should be emphasized that the FCSS, in our hands, provided poor discrimination between MDS and other myeloid clonal diseases.

## 1 INTRODUCTION

Myelodysplastic syndromes (MDSs) cover a wide range of abnormalities, ranging from overt findings such as prominently skewed morphology, ring sideroblasts, and blast excess with or without cytogenetic aberrancies to subtle changes in unilinear MDS patients or patients presenting in very early stages of the disease.^1^ In patients with subtle changes, the diagnosis can be very challenging. In recent years, compiling evidence has emerged for the relevance of flow cytometric assessment as a valuable part of the diagnostic work‐up of MDS. Different approaches have been proposed, many of which are pattern recognition‐based (eg, Wells et al, Chung et al, Huang et al, and Reis‐Alves et al^2^, ^3^, ^4^, ^5^). Pattern recognition provides high specificity and sensitivity but has multiple inherent disadvantages.^6^ Often, it requires highly experienced staff and an extensive panel of antibodies. In addition, if an automated analysis is not applied, it suffers from high inter‐interpreter variation. The high number of parameters included in the evaluation prolongs the analysis and thus lowers the cost‐benefits for a routine diagnostic setting. Some of the methods entail setting up local reference profiles, which also makes it less straightforward to implement (eg, De et al^7^). Other approaches focus on unambiguous criteria, such as expression/no expression of aberrant markers or ratios between cell types.^8^, ^9^, ^10^ This greatly facilitates the implementation in routine diagnostics but at the expense of lower predictive values compared with pattern recognition methods (eg, Satoh et al^8^ vs Chung et al^3^). For this aim, Ogata and his colleagues were pioneers when they suggested 4 cardinal parameters for a flow cytometric score for the diagnosis of MDS with little interexaminer variability.^8^ The 4 cardinal parameters are (1) the percentage of CD34+ myeloid progenitor cells, (2) the frequency of B‐cell precursors within the CD34+ compartment, (3) CD45 expression on myeloid progenitors relative to that on lymphocytes, and (4) the side‐scatter (SSC) properties of neutrophils in comparison with lymphocytes. In 2009, the validation study of the first version of the 4‐point scoring system was published.^11^ In 2012, a multicenter validation study was published evaluating the same system, although reaching slightly different cut‐off values for the respective scores.^12^

In this study, we investigated the accuracy and the feasibility of the Ogata score in our routine diagnostic flow cytometric facility. We suspected the section of samples reaching our lab to be slightly different from those in previous studies. In our setting, the flow cytometry facility is separated from the pathology department, and thus, the choice of panel is based only on the hematologist's sparse requisition notes. This results in a crude pool of samples for which a blast screening is requested. The flow cytometric report will often be completed before the pathologist evaluates the morphology and relates the findings to the patient's clinical history. Thus, the scoring system needs to provide reliable information for all samples submitted to blast screening. We placed special emphasis on identifying necessary changes in the present laboratory practice.

## 2 MATERIALS AND METHODS

### 2.1 Patients and samples

This study was approved by the Danish Data Protection Agency (J no 2008‐58‐0020, case no REG‐126‐2015) and by the Danish Patient Safety Authority (ref. 3‐3013‐1386/1). The latter approval serves as waiver for patient consent according to Danish law. A national public register exists where patients can state their refusal to let biological data enter into research. None of the patients was registered here. All patients with a final diagnosis of MDS fulfilled the criteria of the WHO 2008 classification.

The included cases were selected as unbiasedly as possible, to mimic the normal routine at our hospital in which samples reach the flow cytometric laboratory early in the diagnostic timeline and with only a limited level of diagnostic or clinical information. In practice, we browsed through all requisitions in the period from April 2013 to February 2014 to identify bone marrow samples with any indications of clinical suspicion of MDS or other blast‐related diseases. Clinical suspicion of MDS was most often inferred by unexplained cytopenia. The informative strategy was to exclude other causes such as reactive conditions, nutritional deficiencies, or drug side effects, as well as described by others.^13^ Patients were selected for diagnostic bone marrow sampling when other tests fail to set a clear diagnosis. Typical indication would be unsolved consistent anemia below 10 g/L, thrombocytopenia below 100 billion/L ,and/or neutropenia below 1.8 billion/L. Eighty‐three eligible patient samples were identified. Subsequent exclusion criteria were a) >AML (acute myeloid leukemia) diagnosis (regardless of the actual blast count in the flowcytometric analysis), (b) >2% monoclonal lymphocytes by flow cytometry, and (c) <400 CD34+ events in the collected sample. In total, 35 samples were included in the final assessment.

### 2.2 Flow cytometric analysis

The heparinized samples were kept at ambient temperature and processed within 24 hours following aspiration. The majority of samples were processed between 12 and 24 hours post aspiration.

All samples had been processed using wash‐stain‐lyse procedure. A mastermix containing the following antibodies had been used: 7‐μL CD8 + Lambda‐Fitc/CD56 + Kappa‐PE (Cytognos, cat.no. CYT‐SLPC‐25), 2‐μL CD4‐PerCP‐Cy5.5 (Biolegend, cat no 344608), 3‐μL CD19‐PerCP‐Cy5.5 (Beckman Coulter, cat no A66328), 3.5‐μL CD10‐PC7 (Beckman Coulter, cat no A46527), 2‐μL CD3‐APC (BD Biosciences, cat no 345767), 2‐μL CD14‐APC (BD Biosciences, cat no 345787), 3.5‐μL CD34‐APC‐Alexa Fluor 750 (Beckman Coulter, cat no A89309), 1‐μL CD20‐Pacific Blue (Biolegend, cat no 302328), and 3.5‐μL CD45‐Krome Orange (Beckman Coulter, cat no A96416). Mastermixes were kept at 5°C for a maximum of 28 days. The shelf life for the mastermix has been validated in‐house (data not shown). Thirty‐three microliter bone marrow filtrate adjusted to 10 * 10^9 cells/L (total of approximately 330.000 cells) was washed 3 times in 1 mL BD™ FACSFlow™ (BD Biosciences, cat no 342003) with intermediate 5‐min centrifugations at 300 G. Supernatant was discharged, mastermix was added, and sample incubated 20 minutes in the dark. Erythrocytes were lysed using 3 mL of BD FACS™ lysing solution (BD Biosciences, cat no 349202), after which the sample was centrifuged, separated from supernatant, and added 500‐μL BD™ FACSFlow™. Measurements were performed immediately on a FACSCanto II (BD Biosciences). Approximately 30.000 CD45+ leukocyte events were acquired. The current study was performed by reanalysis of retrospective data. Data files for the included patients were reanalyzed using FACSDiva 8.01 Software (BD Biosciences) in autumn and winter 2015. The mastermix constitutes the standard screening panel in our laboratory. Only some of the markers are necessary for the scoring system, and hence, only those are addressed in this publication.

**Figure 1:**
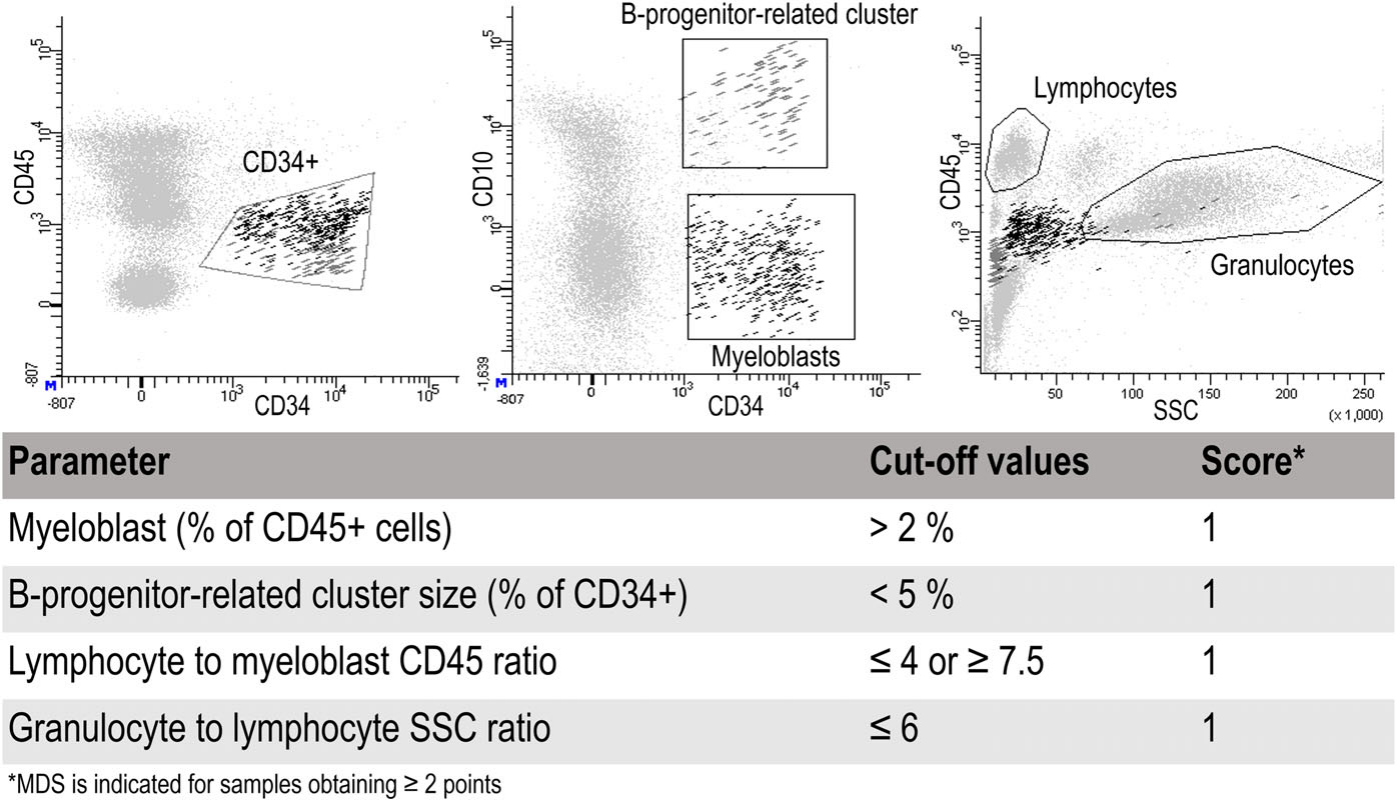
Gates and score calculation. The components of the scoring system are illustrated in representative dot plots from a patient sample. The CD34+ population (left) is divided on the basis of their CD10‐expression into myeloid or B‐progenitor‐related clusters (middle, black and dark gray dots, respectively). CD45/side‐scatter plot (right) shows all singlet events in the analysis and the position of lymphocyte and granulocyte gates. MDS, myelodysplastic syndrome.

Granulocytes and lymphocytes were selected by sideward light scatter (SSC) and CD45 properties. Granulocytes were defined as CD45^dim/int^ SSC^int/high^ and lymphocytes as CD45^bright^SSC^low^. Myeloid progenitor cells were defined as CD45^dim^ in combination with CD34+ and CD10^neg^. B‐cell progenitors were identified by their CD10‐expression, in combination with diminished CD45 and CD34, lower SSC, and FSC properties as compared with myeloid precursors. Each of 4 abnormalities can give a sample 1 point in the Ogata score. A total score of 2 to 4 points indicates MDS. See Figure 1 for details about gating and score calculation.

### 2.3 Pathology assessment

Examination of the bone marrow included an evaluation of bone marrow aspirates and trephine biopsies. Besides this, a peripheral blood film from the patient was examined in parallel. Blast excess was evaluated from a differential count on peripheral blood films and bone marrow aspirates. Dysplasia was assessed for each lineage separately, and only changes above the significance level were reported. Ring sideroblasts were assessed on smears stained with Prussian blue. Clinical information including vitamin deficiencies, drug history, and exposure to toxins were included in the diagnostic considerations. All diagnoses followed the 2008 WHO classification. The Ogata score results were not included in the pathological evaluation of the samples. The majority of patients received a final diagnosis within 2 weeks of bone marrow aspiration. For samples failing to achieve a diagnostic conclusion, a second bone marrow sample was requested after 3 to 6 months. This was the case for 7 samples. After a follow‐up time of at least 18 months, the final diagnoses for the included patients were registered. The diagnoses of 2 patients had been subject to change at the time of follow‐up.

## 3 RESULTS

Seventeen of the 35 included patients received a final diagnosis of MDS, and 4 were diagnosed with other myeloid cancers. For the remaining 14 patients, the bone marrow changes were found to be reactive (see Table 1). All MDS cases correctly received 2 to 4 points in the Ogata flow cytometric scoring systems (FCSS), and all reactive cases correctly received 0 or 1 point. We had 3 false positive MDS calls, all of which were diagnosed with other myeloid cancers (CMML‐1, PV, and PMF). The positive score in the CMML‐1 is expected because the disease entity resembles MDS.^14^ Thus, it is arguable whether the CMML‐1 case should be regarded as a false positive. Here, we attempted to evaluate the Ogata score as an independent diagnostic tool for MDS diagnosis. For this aim, the case represented inaccurate categorization. Only 1 non‐MDS myeloid cancer (a case of ET) was classified correctly as non‐MDS in the FCSS.

**Table 1:** Patient characteristics and score overview

	FCSS Points								
	Myeloblasts	B‐Progenitors	Myeloblast‐CD45	Granulocyte‐SSC	Total Points (1‐4)	Accurate FCSS	True Positive	False Positive	True Negative
Patients correctly diagnosed without need for subsequent samples									
MDS									
MDS with isolated del(5q)	1	1	0	0	2	Yes	x		
MDS/MPN, unclassifiable	1	1	0	0	2	Yes	x		
MDS‐EB1	0	1	1	0	2	Yes	x		
MDS‐EB1	1	1	1	0	3	Yes	x		
MDS‐EB1	1	1	1	0	3	Yes	x		
MDS‐EB2	1	1	1	1	4	Yes	x		
MDS‐EB2	1	1	1	0	3	Yes	x		
MDS	1	1	1	1	4	Yes	x		
CMML1, dysplastic type	0	1	1	1	3	Yes	x		
MDS‐RCUD	0	1	1	0	2	Yes	x		
MDS‐RARS	0	0	1	1	2	Yes	x		
Other myeloid cancer									
ET	0	0	1	0	1	Yes			x
PV	0	1	1	0	2	No		x	
PMF	1	1	0	1	3	No		x	
Reactive changes									
Unspecific reactive changes	0	0	0	0	0	Yes			x
Unspecific reactive changes	0	0	0	0	0	Yes			x
Unspecific reactive changes	0	0	0	0	0	Yes			x
CLL + unspecific reactive changes	0	0	0	0	0	Yes			x
Follicular lymphoma/DLBCL	0	0	1	0	1	Yes			x
MCL	0	0	0	0	0	Yes			x
DLBCL	0	0	0	0	0	Yes			x
Unspecific reactive changes	0	0	1	0	1	Yes			x
Vitamin deficiency	0	1	0	0	1	Yes			x
Unspecific reactive changes	0	1	0	0	1	Yes			x
ICUS	0	0	1	0	1	Yes			x
ICUS	0	0	0	0	0	Yes			x
Patients where subsequent samples were needed for correct diagnosis									
Inconclusive after current sample									
MDS‐EB2	0	0	1	1	2	Yes	x		
MDS/MPN, unclassifiable	1	1	1	0	3	Yes	x		
MDS/MPN, unclassifiable	1	1	0	0	2	Yes	x		
MDS	1	1	1	1	4	Yes	x		
MDS with later progression to mast cell leukemia	1	1	1	1	4	Yes	x		
ICUS	0	0	1	0	1	Yes			x
ICUS	0	0	1	0	1	Yes			x
Incorrectly diagnosed after current sample									
CMML1 (initial diagnosis: MDS)	0	1	1	0	2	No		x	
MDS with later progression to AML (initial diagnosis: likely alcohol‐induced changes)	1	1	1	0	3	Yes	x		

Each included patient is listed with final diagnosis at follow‐up, together with an overview of each awarded FCSS point and the accuracy of the scoring system. FCSS, flow cytometric scoring system; MDS, myelodysplastic syndrome; MPN, myeloproliferative neoplasm; EB, excess blasts; CMML, chronic myelomonocytic leukemia; RT, refractory thrombocytopenia; RCMD, refractory cytopenia with multilineage dysplasia; RARS, refractory anemia with ring sideroblasts; ET, essential thrombocythemia; PV, polycythemia vera; PMF, primary myelofibrosis; CLL, chronic lymphocytic leukemia; DLBCL, diffuse large B‐cell lymphoma; MCL, mantle cell lymphoma; ICUS, idiopathic cytopenia of undetermined significance; AML, acute myeloid leukemia.

## 4 DISCUSSION

A significant role in the diagnostics of MDS has long been anticipated for flow cytometry. Certainly, changes within maturation patterns and protein marker aberrancies can be recognized by this analysis. However, no consensus has yet been reached about the specific approach best suited for the purpose. Several of the suggested FCSS have been dependent on experience of the investigator and prone to high interinterpreter variation.^2^, ^3^, ^4^

Here, we evaluated the feasibility of the 4‐parameter scoring system suggested by Ogata and colleagues^12^ in our routine diagnostic flow cytometric facility. The system is simple and relies on largely objective features. This makes the system an attractive option in laboratories where flow cytometry is separated from other parts of the pathology assessment, or where staff with variable experience perform the flow cytometric interpretation. In our hands, this FCSS was easily implemented without significant additional cost. The required antibodies are already included in our panel for blast screening. We expect a slight rise in the number of diagnostic samples from patients suspected of MDS if the FCSS is implemented as an essential part of the diagnostic work‐up. The number of samples in the series assayed in this study is too small to evaluate sensitivity and specificity, but our results support a high accuracy in the differentiation between reactive bone marrow changes and MDS.

It is worth noticing that all samples which in this study were inconclusive after initial pathology assessment received the correct diagnostic label in the FCSS. In this study, they constituted 20% of the samples. These patients would have undergone additional examinations and experienced prolonged time to diagnosis. The implementation of a reliable scoring system will be of particular benefit for those patients.

The implementation of this scoring system entails some pitfalls. The first challenge to consider is the fact that the scoring system is designed as an evaluation on the bone marrow. However, bone marrow aspirate is subject to potential hemodilution. The degree of hemodilution is not easily determined, and thus, the sensitivity of the system is expected to vary. Several parameters differ from bone marrow to blood, especially in the myeloid compartment. The Ogata score evaluates the degree of granulation on the neutrophilic granulocytes. It was originally suggested to evaluate this parameter on fully mature cells,^11^ but in the final version of the scoring system, this was abandoned because it did not improve the specificity.^12^ Nevertheless, this indicates that a prominent population of fully mature neutrophilic granulocytes from hemodilution should not constitute a problem. Two other parameters are affected by hemodilution, namely, sample content of myeloblasts and B‐cell progenitors. For most low‐risk MDS cases, the subtle blast excess is contained within the bone marrow compartment. Hence, hemodilution will directly dilute the blast % down, thereby increasing the risk of false‐negative FCSS. Likewise, the B‐cell progenitors are not present in the blood. Initially, we decided to rely on the pathologist's evaluation of the purity of the bone marrow sample. All patients with empty coagel were assumed to have massive hemodilution of the marrow aspirate. This resulted in dismissal of 29 out of the 83 samples in our study. For the scoring system to be of practical value in the paraclinic, we needed less stringent exclusion criteria. Instead, we decided to set a threshold of CD34+ cells as exclusion criteria. In order to determine a population in a flow cytometric analysis, we need at least 20 events. Specifically for the 5% cut‐off value for B‐cell progenitor, this would require 400 CD34+ events. These alternative exclusion criteria allowed 11 of the 29 samples originally dismissed, to be included in the study. The inclusion of samples with possible hemodilution will weaken the sensitivity of the scoring system because these samples will have misleadingly low CD34+ events, and thus, less will obtain a point for >2% myeloblasts.

We suggest a practical threshold of 1000 collected CD34+ cells in a patient sample to imply eligibility for the Ogata score evaluation. This would allow quantification of a 5% B‐cell progenitor population (50 events) with a CV of 14%.^15^

**Figure 2:**
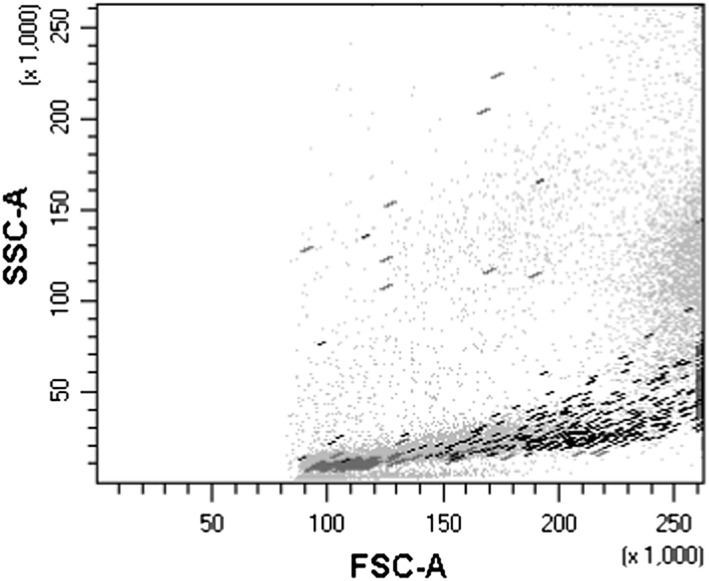
Scatter properties of blast populations. The lymphoblast population (dark gray) has lower forward scatter and lower side‐scatter properties than the myeloblast population (black)

An inadequate threshold in the flow cytometric data collection constitutes another closely related pitfall. The threshold is most often based on forward scatter (FSC) properties. An inadequately high threshold will create a biased loss of B‐progenitor‐related events because these have lower FSC than does the myeloblasts (see Figure 2).

Patients with dominance of monoclonal lymphocytes pose a separate challenge for the FCSS. Aberrant CD45‐expression and/or SSC property are normal features of monoclonal lymphocyte populations.^16^, ^17^ Because the lymphocytes serve as endogenous reference in the FCSS, it is important to assure that the lymphocyte population is normal.

We suggest allowing for minimal infiltration of monoclonal lymphocyte populations because these are not expected to influence the median values of the total lymphocyte population.

The final pitfall in the FCSS is the lack of ability to differentiate between MDS and other clonal diseases of the myeloid lineage. This issue does not have universal relevance. In hospitals where flow cytometry is an incorporated part of pathology examinations, the exclusion of other myeloid neoplasms is ensured up front. In flow cytometric facilities where such close collaboration is not implemented, it can, however, be relevant to consider this. Profound dominance of myeloid blasts in a patient with AML automatically earns the patient the 2 points needed for the MDS label. Even in substandard bone marrow aspirates with deceitfully low blast counts, AML samples will exceed 2% blasts. The myeloblast dominance inevitably outnumbers B‐cell progenitors, thus providing the second point. In our experience, chronic myeloid neoplasms also erroneously seem to trigger the FCSS. All of the 3 patients who in this study obtained a false positive MDS‐label were MPN patients. Indeed, only 1 patient from the MPN group (a case of ET) was scored correctly as non‐MDS. In practice, a positive Ogata score indicates either MDS or another clonal myeloid disease.

### 4.1 Limitations of this study

Our exclusion criterion of 400 CD34+ events has, in practice, excluded all patient samples with less than 1.3% CD34+ cells. Some MDS samples would potentially be in this group due to hemodilution, but it is assumed that the exclusion is biased toward more reactive cases. It is expected that the MDS diagnosis is increasingly difficult to determine with decreasing blast count. Hence, the expected specificity and sensitivity of the scoring system, if implemented with the recommended criteria, is likely to be somewhat poorer than indicated by this study. Furthermore, we cannot exclude the possibility of bias in the initial patient cohort. Not all patients suspected of MDS have their bone marrow assessed by flow cytometry, and the analysis is, for the most part, used merely as a screen for blast excess. The choice of flow cytometric analysis is dependent on the individual physician planning the diagnostic assessment. Thus, there is a risk of bias toward high‐risk MDS in the study cohort. It is well established that the sensitivity of this and other scoring systems is highest for the high‐risk groups.^18^

## 5 CONCLUSION

We find that the Ogata score system for flow cytometric assessment of bone marrows suspected for MDS is feasible to implement, even in small laboratories with no hematopathologists employed. Our findings support the previously reported high accuracy in the segregation of MDS and reactive conditions. However, in our cohort, the system could not distinguish between MDS and other myeloid neoplasms. We suggest a practical threshold for data collection at minimum 1000 CD34+ events. This will limit the effect of hemodilution.

## CONFLICTS OF INTEREST

The authors have no competing interests.

## AUTHOR CONTRIBUTIONS

Conceptualization: SMHM, KK, KKRJ

Funding acquisition: SMHM

Investigation: SMHM, KK, KKRJ

Methodology: SMHM

Resources: SMHM, KK

Writing—original draft: SMHM

Writing—review and editing: SMHM, KK, KKRJ

## References

[1] Garcia‐Manero G . Myelodysplastic syndromes: 2015 update on diagnosis, risk‐stratification and management. Am J Hematol. 2015;90:831‐841. 26294090 10.1002/ajh.24102

[2] Wells DA , Benesch M , Loken MR , et al. Myeloid and monocytic dyspoiesis as determined by flow cytometric scoring in myelodysplastic syndrome correlates with the IPSS and with outcome after hematopoietic stem cell transplantation. Blood. 2003;102(1):394‐403. 12649150 10.1182/blood-2002-09-2768

[3] Chung JW , Park CJ , Cha CH , et al. A combination of CD15/CD10, CD64/CD33, CD16/CD13 or CD11b flow cytometric granulocyte panels is sensitive and specific for diagnosis of myelodysplastic syndrome. Ann Clin Lab Sci. 2012;42(3):271‐280. 22964615

[4] Huang J , Lai P , Zhou M , Weng J , Lu Z , Du X . A multiparametric flow cytometry immunophenotypic scoring system for the diagnosis and prognosis of myelodysplastic syndromes. Clin Lab. 2012;58(11‐12):1241‐1251. 23289195

[5] Reis‐Alves SC , Traina F , Metze K , Lorand‐Metze I . Improving the differential diagnosis between myelodysplastic syndromes and reactive peripheral cytopenias by multiparametric flow cytometry: the role of B‐cell precursors. Diagn Pathol. 2015;10(1):44. 25924846 10.1186/s13000-015-0259-3 PMC4428240

[6] Westers TM , van der Velden VH , Alhan C , et al. Implementation of flow cytometry in the diagnostic work‐up of myelodysplastic syndromes in a multicenter approach: report from the Dutch Working Party on Flow Cytometry in MDS. Leuk Res. 2012;36(4):422‐430. 21982641 10.1016/j.leukres.2011.09.015

[7] De SD , Trullemans F , Jochmans K , et al. Diagnostic potential of CD34+ cell antigen expression in myelodysplastic syndromes. Am J Clin Pathol. 2012;138:732‐743. 23086775 10.1309/AJCPAGVO27RPTOTV

[8] Satoh C , Dan K , Yamashita T , Jo R , Tamura H , Ogata K . Flow cytometric parameters with little interexaminer variability for diagnosing low‐grade myelodysplastic syndromes. Leuk Res. 2008;32(5):699‐707. 17936901 10.1016/j.leukres.2007.08.022

[9] Mathis S , Chapuis N , Debord C , et al. Flow cytometric detection of dyserythropoiesis: a sensitive and powerful diagnostic tool for myelodysplastic syndromes. Leukemia. 2013;27(10):1981‐1987. 23765225 10.1038/leu.2013.178

[10] Xu F , Wu L , He Q , Zhang Z , Chang C , Li X . Immunophenotypic analysis of erythroid dysplasia and its diagnostic application in myelodysplastic syndromes. Intern Med J. 2012;42(4):401‐411. 22032631 10.1111/j.1445-5994.2011.02630.x

[11] Ogata K , Della Porta MG , Malcovati L , et al. Diagnostic utility of flow cytometry in low‐grade myelodysplastic syndromes: a prospective validation study. Haematologica. 2009;94(8):1066‐1074. 19546439 10.3324/haematol.2009.008532 PMC2719029

[12] Della Porta MG , Picone C , Pascutto C , et al. Multicenter validation of a reproducible flow cytometric score for the diagnosis of low‐grade myelodysplastic syndromes: results of a European LeukemiaNET study. Haematologica. 2012;97(8):1209‐1217. 22315489 10.3324/haematol.2011.048421 PMC3409819

[13] Steensma DP . Dysplasia has a differential diagnosis: distinguishing genuine myelodysplastic syndromes (MDS) from mimics, imitators, copycats and impostors. Curr Hematol Malig Rep. 2012;7:310‐320. 23015360 10.1007/s11899-012-0140-3

[14] Swerdlow SH , Campo E , Harris NL , et al. WHO Classification of Tumours of Haematopoietic and Lymphoid Tissues. Revised 4th ed. Lyon: IARC; 2017.

[15] Hedley BD , Keeney M . Technical issues: flow cytometry and rare event analysis. Int J Lab Hematol. 2013;35:344‐350. 23590661 10.1111/ijlh.12068

[16] Carulli G , Cannizzo E , Zucca A , et al. CD45 expression in low‐grade B‐cell non‐Hodgkin's lymphomas. Leuk Res. 2008;32(2):263‐267. 17692374 10.1016/j.leukres.2007.06.002

[17] Sanchez ML , Almeida J , Vidriales B , et al. Incidence of phenotypic aberrations in a series of 467 patients with B chronic lymphoproliferative disorders: basis for the design of specific four‐color stainings to be used for minimal residual disease investigation. Leukemia. 2002;16(8):1460‐1469. 12145686 10.1038/sj.leu.2402584

[18] Bardet V , Wagner‐Ballon O , Guy J , et al. Multicentric study underlining the interest of adding CD5, CD7 and CD56 expression assessment to the flow cytometric Ogata score in myelodysplastic syndromes and myelodysplastic/myeloproliferative neoplasms. Haematologica. 2015;100(4):472‐478. 25637056 10.3324/haematol.2014.112755 PMC4380720

